# Acupuncture for metabolic syndrome: A protocol for systematic review and meta-analysis

**DOI:** 10.1097/MD.0000000000031532

**Published:** 2022-10-28

**Authors:** Rongsheng Jiang, Xiaolin Zhang, Yi Zheng, Long Zhang, Qifan Guan, Zhengri Cong, Yiduo Li, Mingjun Liu

**Affiliations:** a Changchun University of Chinese Medicine, Changchun, China; b Hubei University of Chinese Medicine, Wuhan, China.

**Keywords:** acupuncture, meta-analysis, metabolic syndrome, systematic review

## Abstract

Metabolic syndrome (MS) is showing an epidemic trend worldwide, and its annual incidence is increasing. Conventional treatment options have limitations and it is necessary to develop new management strategies for MS.

**Methods:**

Eligible randomized controlled clinical trials (RCTs) were screened by searching multiple Chinese and English databases. References to the included literature, gray literature in OpenGrey, and other relevant literature, such as clinical studies registered in ClinicalTrials.gov, were also manually searched. Relevant data were extracted, and meta-analysis was performed using Reviewer Manager 5.4.

**Results:**

This study provides a high-quality review of the efficacy and safety of acupuncture in the treatment of MS, and provides a basis for the clinical application of acupuncture in the treatment of MS.

**Conclusion:**

This study provides evidence of the effectiveness and safety of acupuncture in the treatment of MS.

## 1. Introduction

Metabolic syndrome (MS) is characterized by obesity, elevated blood pressure, impaired glucose tolerance, and dyslipidemia.^[1]^ It is a risk factor for diabetes and cardiovascular disease.^[2]^ Data show that 20% to 25% of adults worldwide experience MS, and this figure is on the rise.^[3]^ Recent studies have found that the current global pandemic of novel coronavirus pneumonia (COVID-19) is strongly associated with MS, with hypertension having the strongest association with COVID-19.^[4]^

The current treatment of MS is based on medication^[5,6]^ and lifestyle interventions^[7,8]^ as the main tool. Because of the diversity of MS symptoms, a variety of medications, such as weight loss, hypoglycemia, and antihypertensive medications are used in the treatment plan,^[9]^ the use of these medications has achieved some success. Despite the effectiveness of these medications, side effects, such as drug tolerance, intestinal discomfort, and kidney damage, often occurs. In addition, the long treatment period of MS puts great financial pressure on the patients. Therefore, it is necessary to develop alternative therapies and management strategies for MS.

Acupuncture is widely used as a safe and effective alternative therapy in clinical practice. Numerous clinical studies have shown that acupuncture can alleviate obesity,^[10,11]^ hypertension,^[12,13]^ and diabetes mellitus,^[14,15]^ and has good effects on MS. However, the sample sizes included in these clinical trials are small, and the specific effects of acupuncture in the treatment of MS cannot be accurately assessed.

This systematic review and meta-analysis was conducted to investigate the effectiveness and safety of acupuncture in the treatment of MS by comparing the data of acupuncture combined with drugs and drugs alone to provide a scientific basis for the clinical application of acupuncture in the treatment of MS.

## 2. Materials and methods

This study was analyzed according to the guidelines of the Preferred Reporting Items for Systematic Evaluation and Meta-Analysis (PRISMA)^[16]^ and registered with PROSPERO (registration number: CRD42022360389). This study was analyzed based on the published literature and did not require ethical approval.

### 2.1. Types of studies

Randomized controlled clinical trials (RCTs) without any regional or language restrictions will be included. Duplicates, non-randomized clinical trials, reviews, animal studies, conference abstracts, case reports, and literature for which experimental data were not available were excluded.

### 2.2. Types of participants

We focused on patients diagnosed with MS (age > 18 years). All eligible participants will be included regardless of age, gender, race, nationality, economics, and origin.

### 2.3. Types of intervention

Patients in both the experimental and control groups received the basic treatment provided by the MS treatment guidelines, and the experimental group was treated with acupuncture in addition to the basic treatment. We do not limit the type of acupuncture; patients can receive hand, ear, head, electroacupuncture, or other types of acupuncture therapy. However, to explore the efficacy of acupuncture more clearly, neither the experimental group nor the control group received other types of traditional Chinese medicine (TCM) treatments, such as cupping and moxibustion.

### 2.4. Types of outcome measures

#### 2.4.1. *Main outcome*.

The main purpose of this study was to investigate the efficacy and safety of acupuncture in MS; therefore, we used the efficiency rate as the main outcome of this study. In addition, we selected some representative MS related indicators, including Body mass index, Glycosylated Hemoglobin and Homeostatic Model Assessment for Insulin Resistance.

#### 2.4.2. *Secondary outcomes*.

The secondary outcomes will include TCM symptom score, MS score, and adversarial reactions.

### 2.5. Search strategy

#### 2.5.1. *Electronic search*.

The following databases were searched: PubMed, Cochrane Library, Web of Science, Embase, OVID MEDLINE, China Biology Medicine, Scopus, CNKI, VIP, and Wanfang. The literatures will be searched from the establishment of the database to September 21, 2022, with “Metabolic Syndrome” “Metabolic Syndromes” “Syndrome, Metabolic” “Syndrome X, Metabolic” “Acupuncture Therapy” “Acupuncture Treatment” “Acupuncture Treatments” “Treatment, Acupuncture” “Therapy, Acupuncture” “randomized controlled trial” “randomized” “placebo” and so on were used as keywords for the search. The specific search strategy is presented in Table 1 (PubMed was used as an example).

**Table 1:** Search strategy used in PubMed database.

Number	Search terms
#1	“Metabolic Syndrome” [Mesh]
#2	Metabolic Syndromes [Title/Abstract]
#3	Syndrome, Metabolic [Title/Abstract]
#4	Syndromes, Metabolic [Title/Abstract]
#5	Metabolic Syndrome X [Title/Abstract].
#6	Insulin Resistance Syndrome X [Title/Abstract].
#7	Syndrome X, Metabolic [Title/Abstract]
#8	Syndrome X, Insulin Resistance [Title/Abstract]
#9	Metabolic X Syndrome [Title/Abstract].
#10	Syndrome, Metabolic X [Title/Abstract]
#11	X Syndrome, Metabolic [Title/Abstract]
#12	Dysmetabolic Syndrome X [Title/Abstract]
#13	Syndrome X, Dysmetabolic [Title/Abstract]
#14	#1 or #2 or #3 or #4 or #5 or #6 or #7 or #8 or #9 or #10 or #11 or #12 or #13
#15	“Acupuncture Therapy” [Mesh]
#16	Acupuncture Treatment [Title/Abstract]
#17	Acupuncture Treatments [Title/Abstract]
#18	Treatment, Acupuncture [Title/Abstract]
#19	Therapy, Acupuncture [Title/Abstract]
#20	#15 or #16 or #17 or #18 or #19
#21	randomized controlled trial[Publication Type] OR randomized[Title/Abstract] OR placebo[Title/Abstract]
#22	#14 and #20 and #21

#### 2.5.2. *Manual search of other resources*.

We will search references from the included literature, gray literature in OpenGrey, and other relevant literature, such as clinical studies registered with ClinicalTrials.gov. For ongoing or unpublished RCTs, the authors will be contacted for the latest experimental data.

### 2.6. Literatures collection and organization

**Figure 1.**
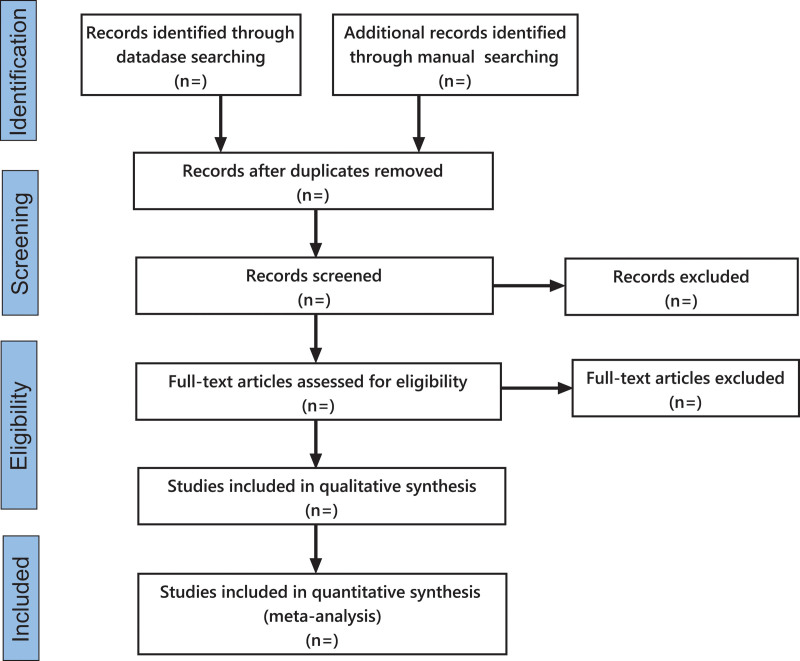
Flow chart of literatures screening.

Two researchers independently performed data collection and organization. The retrieved literatures were first managed by applying NoteExpress to remove duplicate literatures. Two researchers screened the literatures by reading the titles, abstracts, and keywords and recorded the reasons why the literatures were excluded. When the opinions of the two researchers diverge, a third researcher performs the evaluation. The detailed process is illustrated in Figure 1.

### 2.7. Data extraction and export

Two researchers extracted the data from the screened literature and created an Excel spreadsheet. Valid information extracted included the title, author, year, disease diagnosis, sample size, age, gender, intervention modality, treatment period, and outcome indicators. If problems arose, they were resolved through discussion, and a third researcher was invited to participate in the evaluation, if necessary.

### 2.8. Assessment of risk of bias

Two researchers independently assessed the risk of bias in the included studies by using the Cochrane tool. Each study was classified as having high, low, or unclear risk according to the following seven items: random sequence generation, allocation concealment, blinding of participants and personnel, blinding of outcome assessment, incomplete outcome data, selective reporting, and other bias. A score of 1 to 3 was considered high risk, and a score of 4 to 7 was considered low risk. Any disagreement in the process was resolved by a third researcher.

### 2.9. Assessment of heterogeneity

Data heterogeneity will be assessed using the chi-square and *I^2^* tests. When heterogeneity was not significant (*P* ≥ .10 or *I*^2^ ≤ 50%), a fixed-effects model was used for analysis; when heterogeneity was significant (*I*^2^ > 50% or *P* < .10), a random-effects model was used.

### 2.10. Assessment of reporting biases

Assessment of reporting bias will be performed when necessary to confirm the study results. If more than 10 studies were included, the symmetry of the funnel plot was assessed by Begg and Egger tests using Stata 14.0.

### 2.11. *Data synthesis*.

The meta-analysis was performed using Reviewer Manager 5.4. The 95% confidence intervals (CI) were used, mean differences (MD) were calculated for continuous variables, and risk ratios (RR) were calculated for dichotomous variables. Data heterogeneity was assessed using the chi-square and I^2^ tests. When heterogeneity was not significant (*P* ≥ .10, *I*^2^ *≤* 50%), a fixed-effects model was used for analysis; when heterogeneity was significant (*I*^2^ > 50% or *P* < .10), a random-effects model was used.

### 2.12. Subgroup analysis

In cases of significant heterogeneity among the included studies, we performed a subgroup analysis. This will be explored according to age, gender, race, treatment period, sample size, disease category, and other factors that may affect the results.

### 2.13. Sensitivity analysis

To ensure the reliability of the conclusions, we performed a sensitivity analysis based on the method quality, sample size, and missing data. Data analysis and comparison of the results will be performed to assess the reliability of the results.

## 3. Discussion

Clinical evidence suggests that acupuncture has good effects in the treatment of MS^[17,18]^ However, evidence-based medical evidence on the treatment of MS with acupuncture is insufficient, and clinical guidance needs to be improved. Therefore, this study provides a comprehensive analysis of the efficacy and safety of acupuncture in the treatment of MS, as well as an analysis of the shortcomings and prospects of related studies. The results of this study provide a scientific basis for the treatment of MS with acupuncture and facilitate the optimization of MS treatment protocols. This has positive implications for the promotion of acupuncture therapy and the exploration of new management strategies for MS. However, bias and significant heterogeneity may occur in the progress of this study, which may affect the reliability of the research results.

## Author contributions

**Conceptualization:** Rongsheng Jiang, Mingjun Liu.

**Data curation:** Qifan Guan, Zhengri Cong.

**Formal analysis:** Yi Zheng.

**Methodology:** Yiduo Li, Long Zhang.

**Software:** Rongsheng Jiang, Yi Zheng.

**Supervision:** Mingjun Liu.

**Writing** – **original draft:** Qifan Guan, Xiaolin Zhang, Mingjun Liu.

**Writing** – **review & editing:** Rongsheng Jiang.
